# Editorial: Therapeutic Advancements in Psoriasis and Psoriatic Arthritis

**DOI:** 10.3389/fmed.2022.888648

**Published:** 2022-03-29

**Authors:** Saumya Panda

**Affiliations:** Department of Dermatology, Belle Vue Clinic, Kolkata, India

**Keywords:** psoriasis, psoriatic arthritis, therapy, epigenetics, acitretin

Psoriasis and psoriatic arthritis are complex autoimmune diseases affecting about 2–3% of world population. With the advancement in translational research, the pathogenesis of these diseases is better known now compared to a decade ago. New therapeutic targets have been identified, and subsequently more effective therapies are now available for these patients. With these new therapies, psoriatic diseases are much better controlled, and quality of life has improved greatly. Most of these newer therapies are targeting the immune system and their molecular signaling pathways. In this Research Topic, we had planned to gather articles on new therapeutic strategies for psoriatic disease, their limitations and future directions.

We present here a gleaning of contemporary research in this area, encapsulated in 9 articles written by 60 authors. In one of the 3 original articles, Liu et al. explores a novel mechanism of action of acitretin *via* promoting the differentiation of myeloid-derived suppressor cells (MDSC). It is known that increased number of MDSCs are involved in the pathogenesis of psoriasis. Though the role of acitretin as a regulator of keratinocyte proliferation and differentiation is well-known, its effect on immune cells has been less well-understood. This work throws new light on a largely unexplored area.

In another study in this section, Bauer et al. explores epidermal drug delivery through fractional ablative (Er:YAG) laser microporation in a phase I study on plaque-type psoriasis. Topical delivery of etanercept solution to psoriatic plaques *via* laser-generated micropores was found to be generally well-tolerated and safe. The study opens the door to future follow-up studies to find out clinical benefit of this drug delivery system.

Rattanakaemakorn et al. compared a combination of liquid coal tar (liquor carbonis detergens) and 308-nm Excimer lamp with Excimer lamp alone in scalp psoriasis. The combination appeared to have a synergistic effect. This is an important finding in a particularly treatment-resistant site, that not only underscores the importance of an age-old modality like coal tar, but also situates the role of a novel light therapy.

The emergence of proteomics as a technology allows us to have a panoramic view of all potential peptides involved in the interactive pathways operating between cutaneous psoriasis and psoriatic arthropathy, and provides helpful clues as to why a certain subset of cutaneous psoriasis develops arthropathy. Qi et al. has elucidated this aspect in an important mini-review that summarizes the application of proteomics in the development of biomarkers in psoriatic arthritis and identifies possible clinical risk factors in the evolution of psoriatic arthropathy in patients with cutaneous psoriasis.

The role of oxidative stress and that of reactive oxygen species in the pathogenesis of psoriasis is well-known. In an illuminating narrative review, Campione et al. explore the role of dimethyl fumarate (DMF) and its metabolite, monomethyl fumarate, in modulation of pro-inflammatory transcription factors. The comparatively recent association of psoriasis with metabolic syndromes has brought the focus to glutathione-S-transferase dysregulation that is present in obesity, diabetes and cardiovascular disorders. The increase of this enzymatic activity in psoriatic epidermis and its reduction by DMF through formation of covalently linked conjugates is one of the highlights of this review.

In second of the two reviews, Thakur and Mahajan elucidate the therapeutic targets in psoriasis and the novel agents being developed to selectively block or inhibit those targets. Their discussion on the interplay of different epigenetic pathways in pathogenesis of psoriasis and the enzyme inhibitors acting on these pathways make for an illuminating discussion on the novel therapeutic targets in psoriasis.

In an interesting systematic review, Arora et al. deal with the very important issue of combination therapies and manage to come up with some recommendations. They discuss combinations of every kind that have been described in the literature, involving new therapeutic agents (small molecules, biologics), conventional agents and phototherapy.

Gómez-García et al. have done a scoping review of the inhibitors of the Janus kinase–signal transducer and activator of transcription (JAK/STAT) pathway in psoriasis. The application of this class of agents in dermatological disorders is in its infancy. They advocate caution in the interpretation of early phase trials, most of which have been industry-sponsored with a high risk of bias. They also suggest the use of standardized psoriasis-specific outcome measures, which would help reach better decisions.

The last of the three systematic reviews by Zhang et al. is on systematic treatment in nail psoriasis. They recommend to prioritize the use of anti-IL-17 agents in this situation.

To conclude: This Research Topic is a collection of diverse articles providing a gleaning on therapeutic advances in psoriasis and psoriatic arthritis. Through 3 original articles, 1 mini-review, 2 reviews and 3 systematic reviews, a whole lot of new ground, covering pathogenesis of the disease, the interlinking of pathogenetic pathways between cutaneous psoriasis and psoriatic arthritis, new drug delivery systems, systematic reviews of JAK-STAT inhibitors, to name just a few, have been covered by the authors. Many of these subjects are relatively new and/or unexplored, like the role of acitretin in the differentiation of MDSCs, and the role of the latter in the development of severe disease; fractional laser-delivered microporation as a new drug delivery technique in plaque psoriasis; the utilization of proteomics in identifying biomarkers that might be helpful in understanding the subset of cutaneous psoriasis patients who would be at risk for developing psoriatic arthritis, etc. Another important, yet a relatively virgin field of research, highlighted in one of the reviews, is the epigenetic pathways in the pathogenesis of the disease. New light has been thrown on possible mechanisms of action of some agents that are not so new, like fumarates and acitretin. All in all, this bouquet of articles will whet the appetite of anyone who wishes to have a panoramic view of new developments of all aspects of therapy of psoriasis and psoriatic arthritis, particularly if read in conjunction with novel findings in the pathogenesis of both the conditions.

## Author Contributions

The author confirms being the sole contributor of this work and has approved it for publication.

## Conflict of Interest

The author declares that the research was conducted in the absence of any commercial or financial relationships that could be construed as a potential conflict of interest.

## Publisher's Note

All claims expressed in this article are solely those of the authors and do not necessarily represent those of their affiliated organizations, or those of the publisher, the editors and the reviewers. Any product that may be evaluated in this article, or claim that may be made by its manufacturer, is not guaranteed or endorsed by the publisher.

